# Estimation of health risk and economic loss attributable to PM_2.5_ and O_3_ pollution in Jilin Province, China

**DOI:** 10.1038/s41598-023-45062-x

**Published:** 2023-10-18

**Authors:** Yuxia Ma, Yifan Zhang, Wanci Wang, Pengpeng Qin, Heping Li, Haoran Jiao, Jing Wei

**Affiliations:** 1 College of Atmospheric Sciences, Key Laboratory of Semi-Arid Climate Change, Ministry of Education, Lanzhou University, Lanzhou, 730000 China; 2 Meteorological Observatory, Liaoning Provincial Meteorological Bureau, Shenyang, 110000 China; 3 Department of Atmospheric and Oceanic Science, Earth System Science Interdisciplinary Center, University of Maryland, College Park, 20740 USA

**Keywords:** Environmental impact, Risk factors, Health policy

## Abstract

Ambient pollutants, particularly fine particulate matter (PM_2.5_) and ozone (O_3_), pose significant risks to both public health and economic development. In recent years, PM_2.5_ concentration in China has decreased significantly, whereas that of O_3_ has increased rapidly, leading to considerable health risks. In this study, a generalized additive model was employed to establish the relationship of PM_2.5_ and O_3_ exposure with non-accidental mortality across 17 districts and counties in Jilin Province, China, over 2015–2016. The health burden and economic losses attributable to PM_2.5_ and O_3_ were assessed using high-resolution satellite and population data. According to the results, per 10 µg/m^3^ increase in PM_2.5_ and O_3_ concentrations related to an overall relative risk (95% confidence interval) of 1.004 (1.001–1.007) and 1.009 (1.005–1.012), respectively. In general, the spatial distribution of mortality and economic losses was uneven. Throughout the study period, a total of 23,051.274 mortalities and 27,825.015 million Chinese Yuan (CNY) in economic losses were attributed to O_3_ exposure, which considerably surpassing the 5,450.716 mortalities and 6,553,780 million CNY in economic losses attributed to PM_2.5_ exposure. The O_3_-related health risks and economic losses increased by 3.75% and 9.3% from 2015 to 2016, while those linked to PM_2.5_ decreased by 23.33% and 18.7%. Sensitivity analysis results indicated that changes in pollutant concentrations were the major factors affecting mortality rather than baseline mortality and population.

## Introduction

Air pollution, recognized as the largest environmental threat, has led to a massive toll on human health worldwide^1^. Due to the rapid progress of urbanization, industrialization, and energy consumption, China has become one of the countries with the most severe air pollution levels^2^. PM_2.5_ and O_3_, two of the six criteria air pollutants prescribed by the U.S. Environmental Protection Agency, have received considerable attention for their health threats^3,4^. Upon inhalation, PM_2.5_ and O_3_ interact with respiratory tissues, damage lung function, and elicit inflammatory responses, finally leading to various adverse health effects^3,5,6^.

Epidemiological studies have evaluated the health risks related to PM_2.5_ and O_3_ exposure in the United States^7,8^, European^9,10^ and Asian countries^11,12^, such as China. Chen et al. estimated that a 10 μg/m^3^ increment in PM_2.5_ concentration was connected to a 0.13% increase in all-cause mortality in 30 Chinese counties^13^. Moreover, Lu et al. demonstrated that a 10 μg/m^3^ increase in PM_2.5_ concentration led to 0.40% increased non-accidental mortality in several Chinese cities^14^. Similarly, Shi et al. determined that a 10 μg/m^3^ increase in O_3_ concentration contributed to 0.37% increased non-accidental mortality in 128 counties between 2013 and 2018^15^. Vicedo-Cabrera et al. investigated the county-specific effects of O_3_ on mortality and found that a 10 µg/m^3^ rise in O_3_ concentration had a relative risk (RR) (95% confidential interval CI) of 1.001 (1.000–1.003)^16^. In order to tackle air quality issues and safeguard public health, the Chinese government initiated the “Air Pollution Prevention and Control Action Plan” (APPCAP) in 2013. By modifying the energy structure and reducing pollutant emissions, this initiative has succeeded in reducing PM_2.5_ levels in numerous regions. However, PM_2.5_ concentrations in China still exceed the World Health Organization’s (WHO’s) Air Quality Guideline (AQG) recommendations. Moreover, O_3_ pollution has gotten worse contemporaneously^17^. Therefore, air pollution remains a persistent threat to China's public health and economy^18,19^.

Focusing on the United States, Deryugina et al. valued improvements in mortality from reductions in PM_2.5_ concentrations over 1999–2013 at US$24 billion annually^20^. Berman et al. noted that 1,410–2,480 premature deaths could be avoided when the O_3_ reached the standard of 75 ppb^21^. Fann and Risley evaluated that monitored reductions in PM_2.5_ and O_3_ concentrations prevented 22,000–60,000 and 880–4100 premature deaths over 2,000–2007, respectively^22^. Trejo-Gonzalez et al. observed that reducing PM_2.5_ levels to 10 µg/m^3^ prevented 14,666 deaths, leading to savings of US$24.1 billion in Mexico^23^. Lelieveld et al. demonstrated that air pollution caused 790,000 premature deaths per year in Europe^24^. Ballester et al. suggested that controlling PM_2.5_ pollution can prevent thousands of deaths annually, engendering potential benefits^25^. Health-related economic losses have also been assessed in several Chinese cities and regions. For instance, Yao et al. reported that PM_2.5_ and O_3_ exposure carries a substantial health burden and causes substantial economic losses nationwide^26^. Maji et al. estimated that in 2016, the national O_3_-attributable mortality was approximately 74,200, resulting in US$7.6 billion in economic losses^27^. Fan et al. estimated that in the Beijing–Tianjin–Hebei Region (BTH), health hazard–related economic losses due to PM_2.5_ exposure were 122.40 billion CNY, accounting for 1.62% of the region’s GDP (Gross Domestic Product)^2^. Fu et al. reported that in 2015, 2016, and 2017, PM_2.5_ affected 7.41%, 7.05%, and 6.94% of the population in the Central Plains Urban Agglomeration (CPUA), respectively, leading to economic losses of 97.398, 93.516, and 94.485 billion CNY, respectively^28^. Similar studies have been conducted in cities such as Beijing^29^, Shanghai^30^, Guangzhou^31^, Tianjin^32^, and Wuhan^33^. However, most of these studies have focused on the national level or key regions such as BTH, Pearl River Delta (PRD), Yangtze River Delta (YRD), and other major cities, only a few studies have examined the health burden and economic losses at the provincial level.

Therefore, the present study focused on Jilin Province, an underdeveloped area in need of both economic development and environmental protection. We estimated the association of regional PM_2.5_ and O_3_ exposure with mortality in Jilin Province and assessed the resultant health risks and economic losses caused by pollutants. The findings enable to facilitate accurate evaluations of local health risks and losses attributable to pollutants and provide the impetus and scientific basis for enhancing pollution prevention efforts in China.

## Data and methods

### Study area

Jilin Province is located in the geographic center of Northeast Asia and the central part of Northeast China. It spans 121°38′–131°19′E and 40°50′-46°19′N, encompassing an area of approximately 187,400 km^2^. Jilin is a crucial industrial hub in China and had 27.33 million residents at the end of 2016. This province has a temperate continental monsoon climate, with four distinct seasons. The winter is long and cold, with an average temperature below − 11 °C. The summer is short and warm, with an average temperature exceeding 23 °C. The average annual precipitation ranges from 400 to 600 mm. Jilin’s complex geographical conditions result from its proximity to the sea in the east and the Mongolian plateau in the west.

### Data acquisition

#### Data for generalized additive model fitting

**Figure 1:**
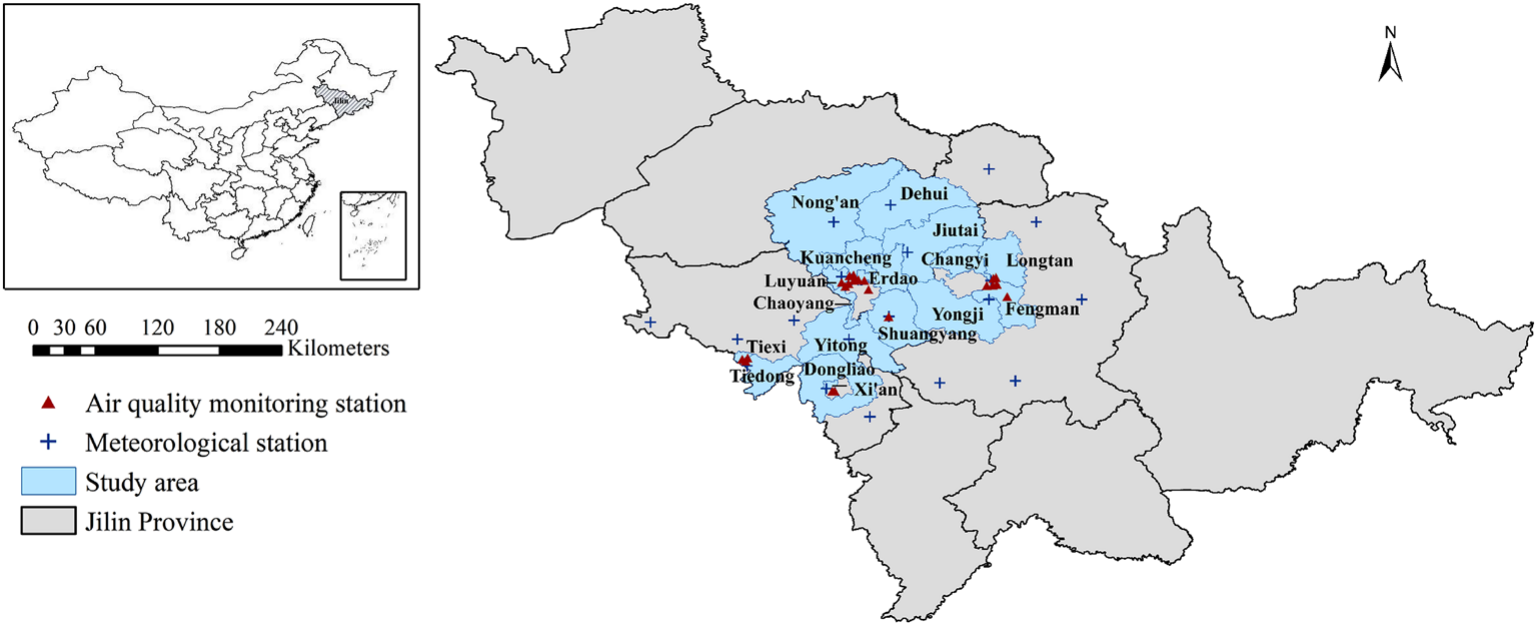
Geographical location of Jilin Province, and distribution of study areas, air quality monitoring stations, and meteorological stations (The map was generated by ArcGIS 10.7 https://www.esri.com/en-us/arcgis/products/arcgis-desktop/resources).

From January 1, 2015, to December 31, 2016, we obtained daily mortality, ambient air pollutant concentration, and meteorological factor data for 17 districts and counties within Jilin Province. The distribution of the study area and monitoring stations is illustrated in Fig. 1.

Mortality data were gathered from the Jilin Provincial Center for Disease Control and Prevention (CDC). These data were reported by district authorities after qualified medical practitioners diagnosed the cause of death and issued death certificates. These data were recorded along with date of death, individual’s age, sex, residence address, and cause of death (classified by the International Classification of Diseases, Tenth Revision [ICD-10]). Data on non-accidental mortality were extracted using the ICD-10 codes A00–R99, and there were no missing data for the study period.

Data on PM_2.5_ and O_3_ concentrations were collected from the China Urban Air Quality Real-time Release Platform (https://air.cnemc.cn:18007). The study area has 21 air quality monitoring stations that are operated throughout the year, with few missing data (< 2.6%). Missing values were filled by linear interpolation of observations from the preceding and subsequent days. These stations were located far from local pollution sources, and adhered to the China Ambient Air Quality Standard, making them representative of urban air pollution levels. For PM_2.5_, we adopted the daily mean concentration, whereas for O_3_, we adopted the daily maximum 8-h moving-average (MDA8) concentration.

Daily meteorological data were obtained from the China Meteorological Data Network (https://data.cma.cn), including average temperature, air pressure, relative humidity (Rh), precipitation, wind speed, and sunshine duration. These data are sourced from national-level surface meteorological stations, which are managed and quality-controlled by the China Meteorological Administration.

As monitoring stations were unevenly distributed over the study area, for districts and counties with multiple monitoring stations, we used the average value of all station observations in the region. For the areas without monitoring stations, the inverse distance weighting (IDW) method was applied to interpolate the observations from stations in other districts or counties in the city (Supplementary Appendix 1).

#### ChinaHighAirPollutants dataset

Data on average annual PM_2.5_ and O_3_ concentrations were obtained from the ChinaHighAirPollutants (CHAP) dataset. These data were utilized to calculate the spatial distribution and total health and economic losses associated with pollutants in Jilin Province. The CHAP dataset comprises a series of full-coverage, long-term, high-resolution, and high-quality datasets of ground-level air pollutants in China, all of which are derived from big data sets (from, for example, ground-based observations, satellite remote sensing products, model simulations, and atmospheric reanalysis) by using artificial intelligence and with data that reflect the spatiotemporal heterogeneity of air pollution^34,35^. Here, the spatial resolution of the PM_2.5_ dataset was 1 × 1 km^2^, with a root-mean-square error (RMSE) of 10.76 μg/m^3^ and a crossvalidation coefficient of determination (CV-R^2^) of 0.92 on a daily basis. Moreover, the spatial resolution of the O_3_ dataset was 10 × 10 km^2^, with an RMSE of 17.10 μg/m^3^ and a CV-R^2^ of 0.87.

#### Population and socioeconomic data

Highly accurate population data in 2015, precise to 1 × 1 km^2^, were provided by the Resource and Environment Science and Data Center (https://www.resdc.cn). These data incorporated various population-relevant characteristics, including land-use type, night light brightness, and settlement density, enabling accurate representation of China’s population distribution. The population distribution in 2016 was calculated using the annual population growth rate of Jilin Province since 2015. Socioeconomic data such as baseline mortality and disposable income in Jilin Province were obtained from the China Statistics Yearbook in 2015 and 2016.

### Statistical methods

#### *Exposure–response relationship of mortality with PM*_*2.5*_* and O*_*3*_

Based on the collected data, we used a generalized additive model to estimate the exposure–response relationship of mortality with PM_2.5_ and O_3_ in 17 districts and counties with different lags. The core model was created as follows:

$${\text{log(E(Y}}_{{\text{i}}} {)) = }\upalpha { + }\upbeta {\text{X}}_{{\text{i }}} {\text{ + s}}\left( {\text{time, df}} \right){\text{ + s}}\left( {{\text{Z}}_{{\text{i}}} {\text{, df}}} \right){\text{ + DOW + Holiday}}$$

where ${\text{E(Y}}_{{\text{i}}} {) }$ refers to the expected non-accidental mortality on day i; $\upalpha$ is the intercept;${\text{X}}_{{\text{i}}}$ indicates PM_2.5_ and O_3_ concentrations;${ }\upbeta$ is the exposure–response coefficient; *s(time, df)* and *s(*${\text{Z}}_{{\text{i}}}$*, df)* refer to the spline function of calendar time and meteorological factors, respectively. *df* is the degrees of freedom; and *DOW* and *Holiday* denote the influence of the week and Chinese public holidays, respectively. The spline function with 3 df was used to eliminate the potential impacts of meteorological factors, and 10 df per year was used to eliminate long-term trends. The results are finally presented as RR with 95% CI per 10 μg/m^3^ increase in PM_2.5_ and O_3_.

We combined the exposure–response coefficient for each district and county through meta-analysis to derive an overall estimate for Jilin Province. A random-effects model (REM) was used to combine the effect estimates when *I*^2^ > 25%; otherwise, the fixed-effects model was selected. The *Mgcv* package in R (version 4.1.2) was used to construct the models, and Stata (version 15.0) was used for the meta-analysis.

#### *Estimates of additional mortality attributable to PM*_*2.5*_* and O*_*3*_

Using geographic information system (GIS) technology, high-resolution pollutant and population data were interpolated into grid with a spatial resolution of 10 km. These interpolated layers were overlaid spatially to calculate the additional mortality in each grid based on the combined exposure–response coefficient. The following equation was used^36,37^:

$$\Delta {\text{Mortality}}_{{\text{i}}} = {\text{Y}}_{0} \times (1 - 1/\exp [\upbeta ({\text{C}}_{{\text{i}}} - {\text{C}}_{0} )]) \times {\text{POP}}_{{\text{i}}}$$

where $\Delta {\text{Mortality}}_{{\text{i}}}$ is the additional mortality caused by PM_2.5_ and O_3_ in grid i, ${\text{Y}}_{{0}}$ is baseline mortality, $\upbeta$ is the combined exposure–response coefficient, ${\text{C}}_{{\text{i}}}$ is the average annual pollutant concentration at grid i,${\text{ C}}_{{0}}$ is the threshold concentration below which no health risk is assumed, and ${\text{ POP}}_{{\text{i}}}$ is the exposed population. Because it has been reported that there appeared to be no concentration (or threshold) beneath which adverse events were not observed^38^. 0 μg/m^3^ was chosen as the threshold for pollutant concentrations here.

After calculating the mortality in each grid separately, we performed spatial interpolation to obtain the distribution of the average annual additional mortality across Jilin Province and calculated the total death count using the spatial analyst tool in ArcMap (version 10.3).

#### Estimation of economic losses

Economic losses associated with additional mortality were quantified using the value of a statistical life (VSL). VSL is calculated through survey studies, reflecting the value people attach to a slight reduction in mortality risk ^39^. Given that VSL is affected by individual income levels, it varies across provinces and years. In this study, we applied a base VSL estimate of 1.68 million CNY derived from Xie’s study in Beijing^40^. The estimated unit economic losses in Jilin Province was converted using the following Eq. ^19,36^:

$${\text{VSL}}_{2015/2016} = {\text{ VSL}}_{base} \times \left( {\frac{{{\text{Income}}_{{{\text{Jilin}}, 2015/2016}} }}{{{\text{Income}}_{{{\text{base}}, 2010}} }}} \right)^{e}$$

where ${\text{VSL}}_{{2015/2016}}$ is the unit loss per case in Jilin Province in 2015 and 2016; ${\text{VSL}}_{{{\text{base}}}}$ is 1.68 million CNY; ${\text{Income}}_{{{\text{Jilin}}, 2015/2016}}$ and ${\text{Income}}_{{{\text{base}}, 2010}}$ is the per capita disposable income of Jilin and Beijing at the given time, respectively; and *e* is the income elastic coefficient, set at 0.8 according to the Organisation for Economic Co-operation and Development (OECD) recommendations^41^.

Based on the calculated unit loss, the economic losses in each grid were estimated by the following equation:

$${\text{E}}_{{\text{i}}} = {\text{VSL}}_{2015/2016} \times \Delta {\text{Mortality}}_{{\text{i}}}$$

The spatial distribution and total economic losses were also obtained using ArcMap (version 10.3).

### Sensitivity analysis

Because additional mortality is influenced not only by changes in pollutant concentrations but also by social factors such as baseline mortality and population, we quantified the effect of each variable on health and calculated its relative contribution through sensitivity analysis—where only one variable was allowed to change at a time from 2015 to 2016, whereas the other variables were kept constant^42^.

## Results

**Figure 2:**
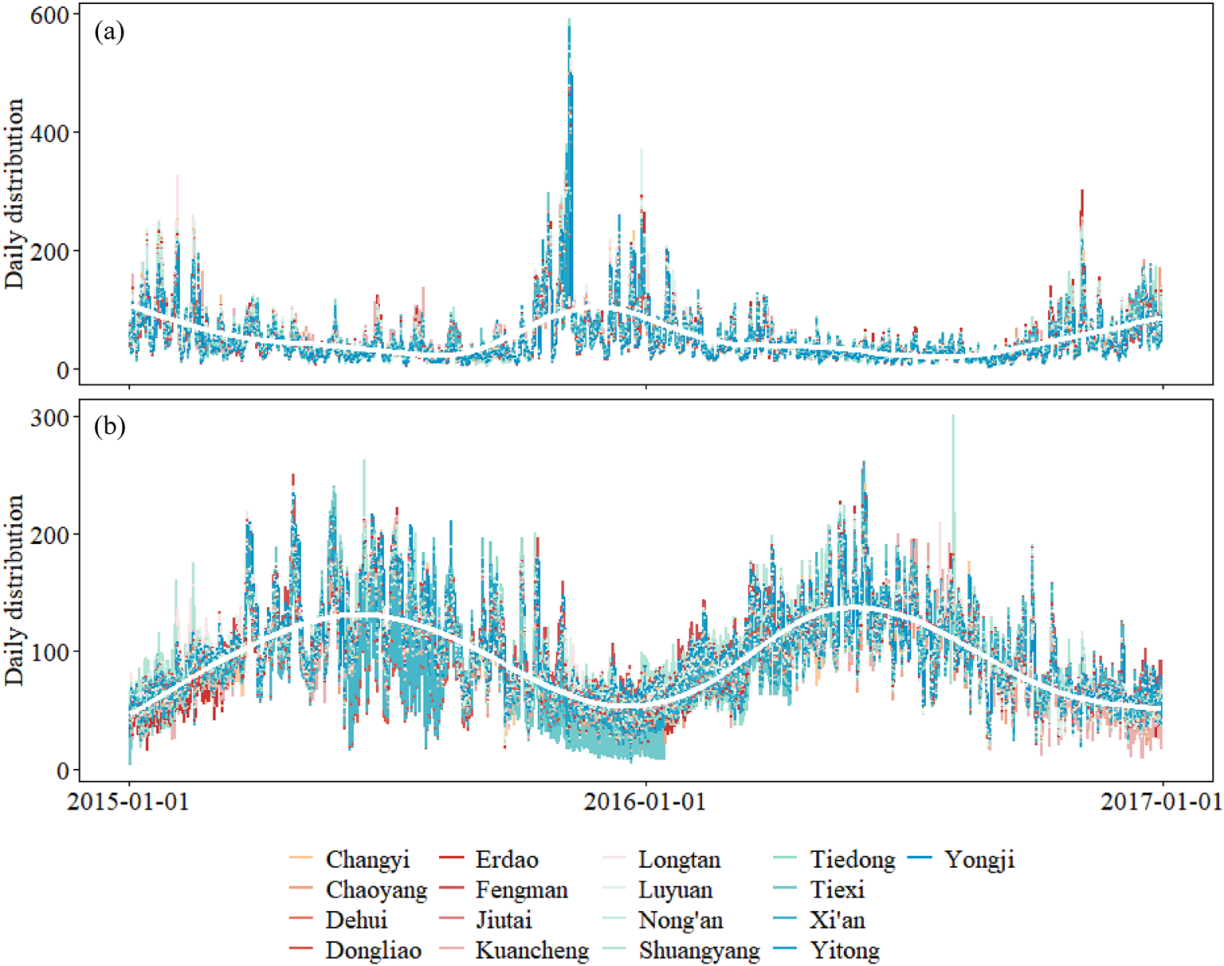
Time series of PM_2.5_ (**a**) and O_3_ (**b**) in 17 districts of Jilin Province over 2015–2016.

During 2015–2016, 83,868 non-accidental deaths (48,961 male and 34,907 female deaths) were reported across 17 districts in Jilin Province. The daily characteristics of the mortality, air pollutants, and meteorological factors were summarized in Table 1. The average daily mortality ranged from 3.3 to 14.6, with the lowest value observed in Xi’an and the highest value in Dehui. Daily PM_2.5_ concentration ranged from 41.2 to 58.6 μg/m^3^, exceeding the WHO's recommended limit of 15 μg/m^3^. The MDA8 of O_3_ ranged from 84.5 (in Chaoyang) to 110.3 μg/m^3^ (in Shuangyang). Meteorological conditions, including average daily mean temperature (ranged from 6.1 °C to 7.6 °C) and relative humidity (ranged from 61.7% to 68.3%), were fairly consistent across the study area, reflecting the temperate monsoon climate in Northeast China. Furthermore, the concentrations of PM_2.5_ and O_3_ showed distinct seasonal patterns, with PM_2.5_ peaking in autumn and winter and O_3_ peaking in summer (Fig. 2).

**Table 1:** Descriptive statistics of daily mortality, pollutants, and meteorological factors in 17 districts of Jilin Province over 2015–2016.

District	Daily mortality	Pollutants ($\upmu$ g/m^3^)		Meteorological factors	
		PM_2.5_	O_3_	Temperature (°C)	Relative humidity (%)
Changyi	8.6	46.6	94.6	7.0	62.2
Chaoyang	9.6	54.0	84.5	7.1	62.1
Dehui	14.6	55.2	92.4	6.1	66.0
Dongliao	4.2	51.7	88.9	6.7	65.9
Erdao	6.3	58.6	90.0	7.0	62.7
Fengman	4.6	41.2	106.2	6.9	62.7
Jiutai	7.1	55.1	92.6	6.1	68.3
Kuancheng	6.8	56.7	87.3	7.0	62.5
Longtan	6.3	49.0	102.2	6.9	62.6
Luyuan	7.5	58.5	97.4	7.2	61.7
Nong'an	9.3	54.4	93.9	6.5	66.1
Shuangyang	6.3	49.6	110.3	6.6	66.4
Tiedong	5.4	55.0	87.2	7.6	62.8
Tiexi	3.9	50.6	90.3	7.6	62.6
Xi'an	3.3	51.9	88.7	6.7	65.9
Yitong	4.9	53.5	88.2	6.7	67.3
Yongji	6.2	45.5	103.0	6.5	65.9

**Figure 3:**
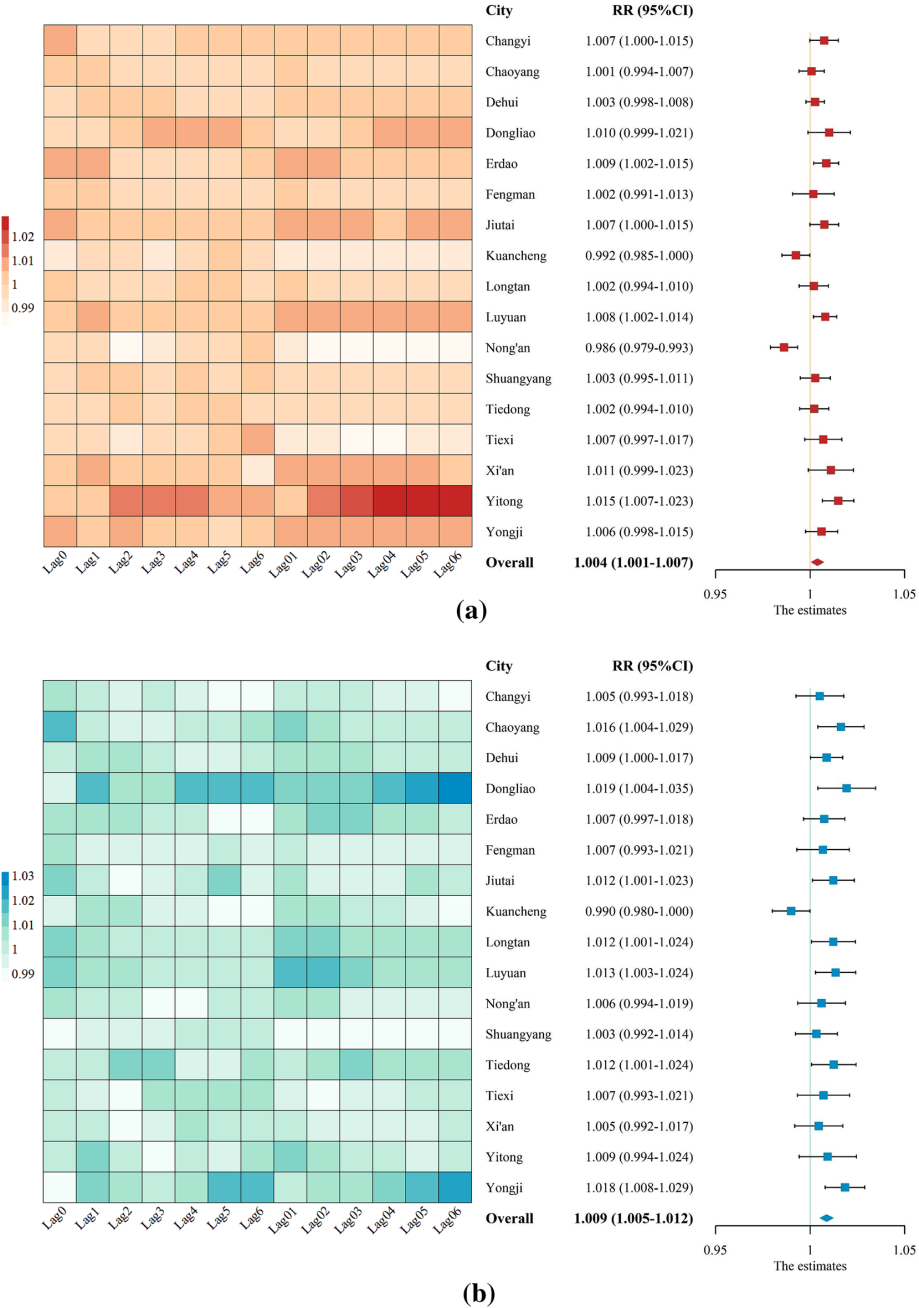
RRs (95% CIs) of mortality associated with 10 μg/m^3^ increases in PM_2.5_ (**a**) and O_3_ (**b**) concentrations at different lag days in 17 districts of Jilin and the combined estimates obtained from meta-analysis.

Figure 3 illustrates the estimated effects of PM_2.5_ and O_3_ on non-accidental mortality at different lag days. The RRs of mortality associated with per 10 μg/m^3^ increase in PM_2.5_ and O_3_ varied by districts. The maximum RR (95% CI) for PM_2.5_ during the single-day lag ranged from 0.986 (0.979–0.993) to 1.015 (1.007–1.023). The strongest and smallest effect was estimated in Yitong and Nong’an respectively. Significant PM_2.5_-mortality associations were only found in Changyi, Erdao, Jiutai, Luyuan, and Yitong. For O_3,_ the maximum RR (95% CI) ranged from 0.990 (0.980–1.000) to 1.019 (1.004–1.035), with Dongliao having the highest impact and Kuancheng the lowest. Significant O_3_-mortality associations were observed in Chaoyang, Dehui, Dongliao, Jiutai, Longtan, Luyuan, Tiedong, and Yongji.

Given the heterogeneity among districts, REM was used to combine the effect estimates for both PM_2.5_ and O_3_. The results demonstrated that the overall effect of the two pollutants was significant, with O_3_ having a more substantial impact than PM_2.5_. On average, every 10 µg/m^3^ increase in PM_2.5_ and O_3_ concentrations corresponded to overall RRs (95% CI) of 1.004 (1.001–1.007) and 1.009 (1.005–1.012), respectively.

**Figure 4:**
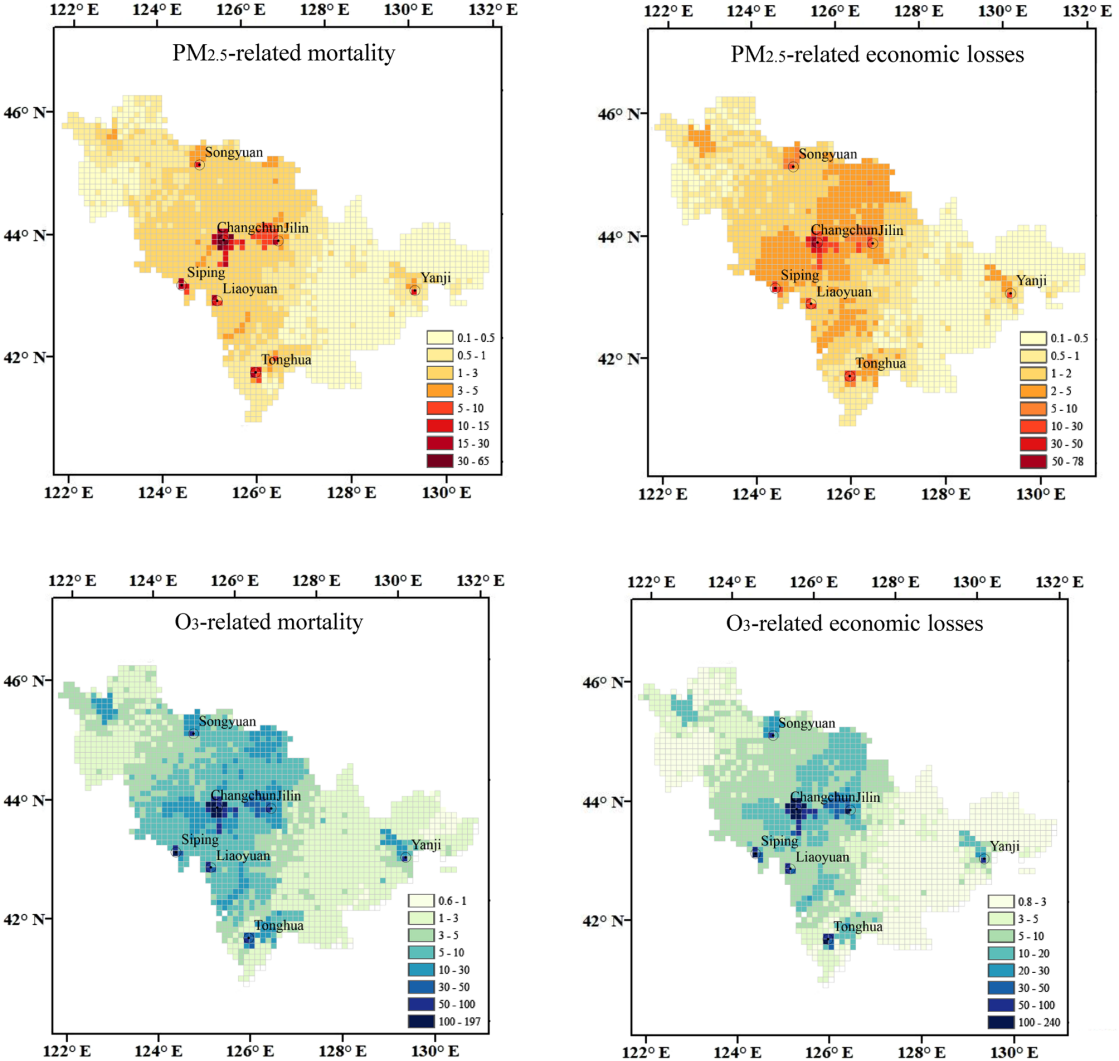
Spatial distribution of the average annual non-accidental mortality (left) and economic loss (right) associated with PM_2.5_ and O_3_ exposure in Jilin Province over 2015–2016 (unit for mortality: person; unit for economic loss: million CNY) (The map was generated by ArcGIS 10.7 https://www.esri.com/en-us/arcgis/products/arcgis-desktop/resources).

The average annual additional mortality and economic losses associated with PM_2.5_ and O_3_ exposure in Jilin Province varied across regions due to uneven spatial distribution of pollutants and population. As shown in Fig. 4, areas heavily affected by PM_2.5_ and O_3_ were concentrated in the central regions of Jilin, with high-value areas clustered in urban centers and sparsely distributed in suburban areas. Changchun, the provincial capital, has high levels of pollutants and high population density, leading to severe health and economic losses. Other cities, such as Songyuan, Jilin, Yanji, Siping, Liaoyuan, and Tonghua, also experienced significant economic losses. Province-wide, the average annual additional mortality and economic losses attributable to O_3_ exposure were approximately four times higher than those attributable to PM_2.5_ exposure.

**Figure 5:**
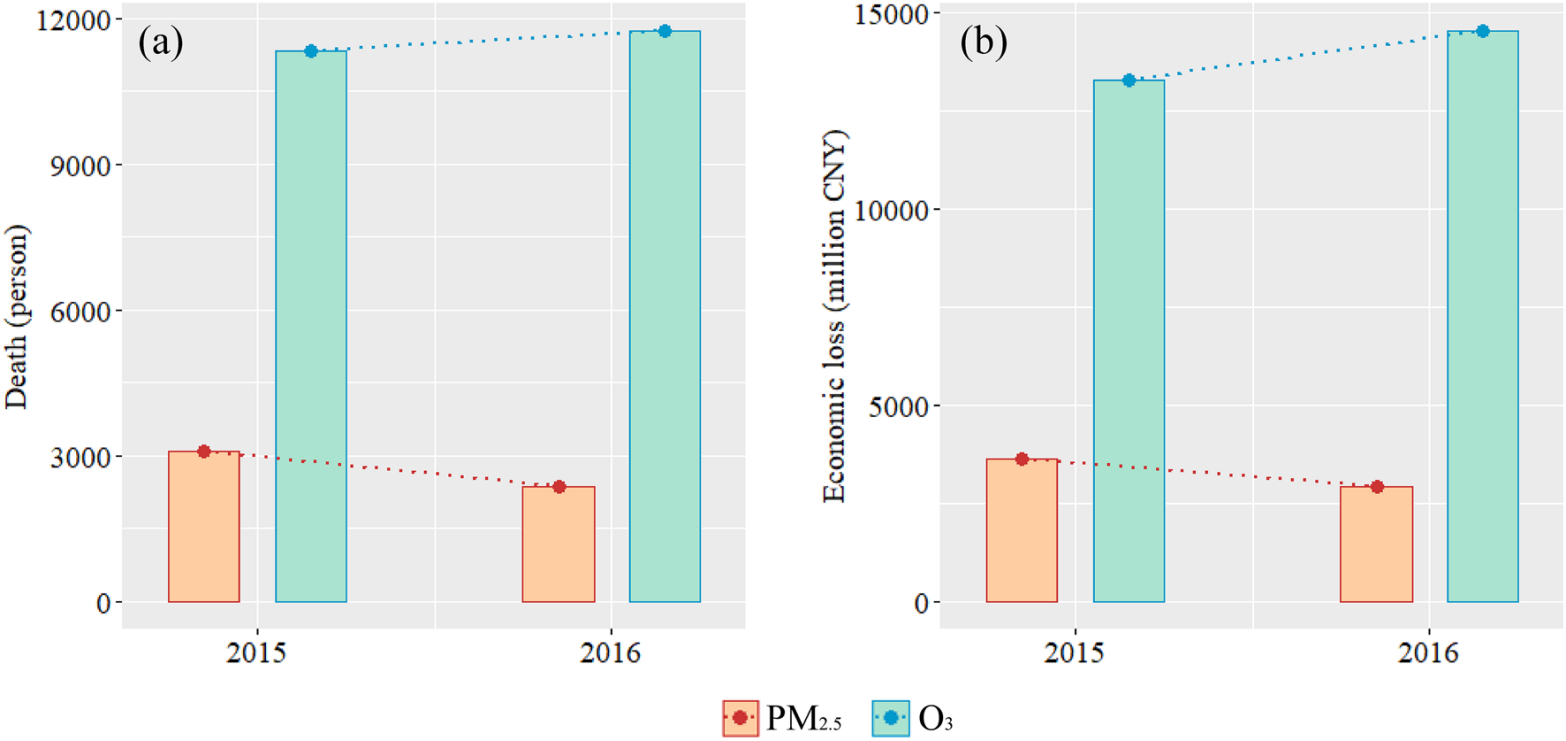
Total non-accidental mortality (**a**) and economic loss (**b**) associated with PM_2.5_ and O_3_ exposure in Jilin Province in 2015 and 2016.

Figure 5 presents the total additional mortality and economic losses in Jilin Province over 2015–2016. In 2015, PM_2.5_ exposure led to a total of 3,077.971 (95% CI 795.073–5,481.245) additional deaths and economic losses of 3,614.971 (95% CI 933.786–6,437.533) million CNY, which decreased to 2,372.745 (95% CI 611.900–4,232.761) additional deaths and 2,938.809 (95% CI 757.881–5,242.568) million CNY in economic losses in 2016. Conversely, O_3_ exposure caused 11,318.892 (95% CI 6,608.547–15,395.248) additional deaths and economic losses of 13,293.649 (95% CI 7761.511–18,081.189) million CNY in 2015, which increased to 11,732.382 (95% CI 6856.330–15,944.577) additional deaths and 14,531.366 (95% CI 8492.039–19,748.460) million CNY in economic losses in 2016.

The sensitivity analysis revealed that the changes in additional mortality between 2015 and 2016 were influenced by pollutant concentrations, baseline mortality, and population. The number of deaths attributable to PM_2.5_ exposure decreased by 23.33% in 2016 compared to 2015. This decrease was primarily driven by a reduction in PM_2.5_ concentrations (21.39%), with smaller contributions from changes in baseline mortality (1.21%) and population (0.73%). In contrast, the number of deaths attributable to O_3_ exposure increased by 3.75% in 2016 compared to 2015. This increase was primarily due to a rise in O_3_ concentration (5.69%), partially offset by changes in baseline mortality (1.21%) and population (0.73%) (Table 2).

**Table 2:** Changes in additional mortality over 2015–2016 due to separate changes in pollutant concentration, baseline mortality, and population.

	Variable	Change in additional mortality	Relative contribution (%)*
PM_2.5_	Concentration	− 658.527	− 21.39%
	Baseline mortality	− 37.319	− 1.21%
	Population	− 22.361	− 0.73%
O_3_	Concentration	644.399	5.69%
	Baseline mortality	− 137.239	− 1.21%
	Population	− 82.230	− 0.73%

*Calculated as [(2016 data − 2015 data)/2015 data] × 100%.

## Discussion

In this study, we assessed the health burden and economic losses associated with PM_2.5_ and O_3_ exposure in Jilin Province over 2015–2016. The results indicated that despite the implementation of China’s pollution prevention and control policies has facilitated air pollutant hazard mitigation, PM_2.5_ and O_3_ still pose a significant health risk and constitute a source of economic losses in Jilin Province.

PM_2.5_ and O_3_ stimulate oxidative stress, promote airway hyperresponsiveness, induce airway inflammation, and affect vascular endothelial function and thus influence heart rate and vascular tone regulation^43,44^. Numerous studies have well revealed the exposure–response relationship of non-accidental mortality with PM_2.5_ and O_3_. Cai et al. observed that for every 10 μg/m^3^ increase in PM_2.5_ concentration, the strongest excess risk (ER; 95% CI) for non-accidental mortality was 0.67% (0.01%–1.33%) in Shenzhen^45^. Xu et al. reported 0.17% (95% CI 0.05%–0.29%) increased non-accidental mortality in Beijing^46^, and Fang et al. observed 0.26%–0.35% increased mortality in Shanghai^47^. Li et al. found that a 10 μg/m^3^ increase in O_3_ concentration is correlated with a 0.56% (95% CI 0.36%–0.76%) higher risk of non-accidental death in Guangzhou^42^. Kan et al. reported a 0.31% (95% CI 0.04%–0.58%) increase in mortality in Shanghai^48^, whereas Lei et al. reported 0.05% (95% CI 0.42%–0.53%) increased mortality in Hefei^49^. In the present study, the maximum RR (95% CI) during the single-day lag ranged from 0.986 (0.979–0.993) to 1.015 (1.007–1.023) for PM_2.5_ and from 0.990 (0.980–1.000) to 1.019 (1.004–1.035) for O_3_. The effect estimates vary between counties and regions because of differences in pollution conditions, population characteristics, and lifestyles^50^. After the effect values were combined in a meta-analysis, both pollutants were found to be significantly associated with non-accidental mortality, with an overall RR (95% CI) of 1.004 (1.001–1.007) and 1.009 (1.005–1.012), respectively.

Our study revealed an uneven spatial distribution of additional mortality and economic losses associated with PM_2.5_ and O_3_ in Jilin Province. The results demonstrated a higher health burden in the central region and a lower health burden in the western region. The central region, characterized by large cities, had higher pollutant concentrations and population density, so people are more likely to be subjected to a higher level of pollutants^51^. In contrast, the western region, closer to the coastline, experienced improved pollution diffusion and better air quality^52^. As the capital city of Jilin Province, Changchun bore the most severe health burden and economic losses. Another study in Gansu Province also reported that economic costs in the capital city are higher than those in other cities^53^. Provincial capitals are typically demonstrating a rapid increase in population, urbanization, industrialization, and energy consumption. Thus, high emission, a developed economy, and high population density result in large health burden and economic losses.

In 2015, PM_2.5_ exposure resulted in 3,077.971 additional deaths and 3,614.971 million CNY in economic losses, accounting for 0.26% of the province’s GDP. However, in 2016, these figures decreased to 2,372.745 deaths and 2,938.809 million CNY in economic losses, respectively, accounting for 0.20% of GDP. At the national level, Sun et al. reported that in China, PM_2.5_ affected an average of 15.03 million people per year and caused economic losses of 1.71% of China's GDP, about US$86,886.94 million^54^. Diao et al. found that in China’s 338 cities, the overall economic losses caused by exposure to PM_2.5_ in 2015 was 1.846 trillion CNY, which was 2.73% of their total annual GDP^51^. Chen et al. calculated in 2014, when the PM_2.5_ reached the national air quality standards in China, an estimated 0.35 million deaths were avoided, along with an economic gain of 430 billion CNY^37^. Fan et al. estimated that in 2016, the average economic cost caused by PM_2.5_ was 122.40 billion CNY in the BTH, accounting for 1.62% of the region’s GDP^2^. Wang et al. explored that in the YRD, the short-term mortality and economic losses due to PM_2.5_ were estimated to be 13,162 and 22.1 billion CNY in 2010, respectively^55^. Similar results have also been drawn from studies in the PRD and the Fenwei Plain (FWP)^56,57^. At the provincial and city levels, Gao et al. found that PM_2.5_ exposure contributed 17.6% to premature mortality in Hebei Province^19^. Liao et al. evaluated the health burden and economic losses in the cities of Gansu Province and reported that from 2015 to 2017, PM_2.5_ caused 1,644,870, 1,551,447, and 1,531,372 deaths, respectively, with an economic loss of 42,699, 43,982 and 44,261 million CNY, respectively^53^. Cui et al. demonstrated that decreasing PM_2.5_ concentrations to 15 μg/m^3^ may reduce mortality and morbidity by 70% and 95%, respectively, in Jinan, saving US$1,289.5 million^58^. Similar studies have been conducted in Guangzhou^31^, Shanghai^30^, Lanzhou^59^, and Taiyuan^60^. Although these studies are not directly comparable for the differences in study areas, study design and period, health endpoints, and population lifestyle, their results are valid. In addition, over 2015–2016, the losses caused by PM_2.5_ exposure exhibited a downward trend, as noted previously. Ding et al. estimated the change in PM_2.5_-related mortality from 2013 to 2017 and indicated that the estimated mortality in China decreased significantly from 1.389 (95% CI 1.005–1.631) million CNY to 1.102 (95% CI 0.755–1.337) million CNY^61^. Fu et al. observed that the health damage caused by PM_2.5_ exposure decreased in the CPUA between 2015 and 2017^28^. Cui et al. discovered significant decreases in ambient PM_2.5_ concentrations in Jinan from 2013 to 2017, which prevented 2,317 premature deaths and saved US$317.7 million^58^. In Beijing and Wuhan, the worsening trend of air quality began to reverse as well^33^. These results jointly indicate that the implementation of APPCAP in China has yielded substantial environmental, health, and economic benefits.

Along with the decrease in PM_2.5_ concentrations, however, O_3_ concentrations have been gradually increasing in recent years, leading to higher additional mortality and economic losses. Gao et al. found that over 2015–2017, in Handan, Hebei Province, the average annual O_3_ concentration increased by 35.1%, whereas PM_2.5_ concentration decreased by 6.6%^3^. Zhang et al. reported that in 331 cities in China, the rate of the population exposed to O_3_ increased from 13.35% in 2015 to 14.15% in 2020, whereas that of the population exposed to PM_2.5_ declined over 2015–2020^62^. Guan et al. revealed that in BTH and FWP cities, the PM_2.5_-related health impact decreased by 17.14% but the O_3_-related health impact increased by > 90% between 2015 and 2020^4^. Wang et al. revealed that in 74 Chinese cities, the number of additional deaths correlated with short-term and long-term PM_2.5_ exposure decreased by approximately 72.49% and 10%, respectively, while O_3_-related deaths increased by 76.16% and 130.57%, respectively, during 2013–2018^63^. Our study in Jilin Province also demonstrated that O_3_ exposure contributed to an increase in mortality and economic losses. In 2015, O_3_ exposure contributed to 11,318.892 (95% CI 6,608.547–15,395.248) additional deaths and 13,293.649 (95% CI 7,761.511–18,081.189) million CNY in economic losses, accounting for 0.95% of the province’s GDP. In 2016, these figures rose to 11,732.382 (95% CI 6,856.330–15,944.577) and 14,531.366 (95% CI 8,492.039–19,748.460), respectively, accounting for 0.98% of the province’s GDP. The increasing trends for O_3_ in China after air pollution control may be because the decreases in PM_2.5_ further affected O_3_ concentration through changes in aerosol chemistry and photolysis rates^64,65^. In the future, comprehensive air pollution control strategies should be implemented on the basis of existing policies.

Additional mortality is simultaneously affected by pollutant concentrations, baseline mortality, and population. For mortality attributable to PM_2.5_ exposure, these factors collectively contributed to a decrease in mortality, with PM_2.5_ concentration changes being the key driver. For mortality attributable to O_3_ exposure, changes in O_3_ concentration resulted in increased mortality, although changes in baseline mortality and population partially offset this increase. In the literature, Li et al. assessed premature mortality in China with respect to independent variations in PM_2.5_, baseline mortality, and population^42^. The authors noted that PM_2.5_ change had a greater effect on mortality than other factors did and that it was the primary determinant of changes in mortality. Yin et al. reported that between 1990 and 2017, a decline in air pollution exposure partially offset population growth and aging in China^66^. Zhang et al. quantified the contribution of different factors to the health burden and noted that the reduction in pollutant concentration is the dominant factor in mitigating adverse health effects^67^. Consequently, from a risk perspective, reducing pollutant concentrations is likely the most effective strategy for reducing health risks associated with ambient pollutants.

Our study has some limitations. The first major limitation is related to safety thresholds for air pollutants. At present, no consistent indications for setting a specific threshold have been reported. In the present study, we used 0 μg/m^3^ as the threshold for both PM_2.5_ and O_3_ concentrations, which may yield a relatively higher estimation than studies set thresholds according to China’s national standards and WHO standards^63,68^. Moreover, we estimated the spatial distribution of health burden and economic losses with a 10-km resolution using GIS. In this process, PM_2.5_ and population data with a 1-km resolution were converted to an identical resolution, which may potentially obscure or overestimate the benefit estimates^69^. Finally, when calculating health burden and economic losses, we assumed a static population in the study region even though actual exposure is typically dynamic, which may result in underestimated results^70^. In addition, other factors such as age structures, income conditions, and medical conditions in different regions were not considered during the current evaluation, affecting the current assessment accuracy. Despite these limitations, the overall trend identified in this study remained unaffected, and these findings can still aid in assessments of the actual situation in Jilin Province.

## Conclusion

According to our findings, although the air quality has improved in recent years, Jilin Province continues to face significant health and economic challenges associated with PM_2.5_ and O_3_ exposure. During the study period, the additional mortality and economic losses related to PM_2.5_ exposure were significantly lower than those related to O_3_ exposure. Specifically, from 2015 to 2016, the health impact and economic losses attributable to PM_2.5_ exposure decreased by 23.33% and 18.7%, respectively, whereas those linked to O_3_ exposure increased by 3.75% and 9.3%, respectively. Pollutant concentration is the dominant factor affecting mortality. Therefore, more aggressive air pollution control strategies should continue to be implemented in the future to reduce ambient PM_2.5_ concentrations and mitigate O_3_ pollution.

### Supplementary Information

Supplementary Information.

## Data Availability

The datasets are not publicly available due to data privacy but are available from the corresponding author on reasonable request.
