# Supplementary material for: Estimation of health risk and economic loss attributable to PM2.5 and O3 pollution in Jilin Province, China
Source: Sci Rep. 2023 Oct 18;13:17717. doi: 10.1038/s41598-023-45062-x (PMC10584970; doi:10.1038/s41598-023-45062-x)
Supplement: Supplementary file 1 — Supplementary Information. [file 41598_2023_45062_MOESM1_ESM.docx]

**Supplementary Information**

**Appendix 1. IDW Method**

IDW is a spatial interpolation method that takes distance into account as a weight. When predicting the target point, it assumes that the sample values closer to the target point location have a greater effect on the interpolated value than those further away, and thus the measurement points that are closer to the target point are assigned a higher weight. The calculation formula is as follows:

$$\text{Z=}\frac{\sum_{\text{i=1}}^{\text{n}} \frac{\text{Z}_{\text{i}}}{\text{d}_{\text{i}}^{\text{k}}}}{\sum_{\text{i=1}}^{\text{n}} \frac{\text{1}}{\text{d}_{\text{i}}^{\text{k}}}}$$

Where Z is the interpolated value to be calculated at the target point; n is the number of known observation samples with measurements surrounding;$\text{Z}_{\text{i}}$ is the observation value of the i th known sample;$\text{d}_{\text{i}}$ is the distance between the target point and the i th sample point in the area; and k is the specified exponent, which defaults to 2.
